# Fibroid Phthisis in the Gold Mines

**Published:** 1899-09-23

**Authors:** 


### FIBROID PHTHISIS IN THE GOLD MINES.

At a recent meeting of the Rocky Mountain Inter-State Medical Association, Dr. Betts, of Salt Lake City, drew attention to the prevalence of lung trouble among those engaged in the mills where certain gold-bearing ores are reduced to fine powder. In these mills the air was constantly filled with an impalpable dust, and the results were disastrous. The average time the men were able to continue working was 13 months, and the average time they survived after leaving the mill was 10 months.

